# Integrating bioinformatics to explore HPV-31 and HPV-52 E6/E7 proteins: from structural analysis to antigenic epitope prediction

**DOI:** 10.3389/fimmu.2025.1561572

**Published:** 2025-07-25

**Authors:** Qixue Cai, Yifan Feng, Wenbo Dong, Yanling Meng

**Affiliations:** ^1^ Department of Pulmonary and Critical Care Medicine, Institute of Respiratory Disease, The First Hospital of China Medical University, Shenyang, Liaoning, China; ^2^ Department of Gastrointestinal Surgery, The First Hospital of China Medical University, Shenyang, Liaoning, China; ^3^ The First Clinical College, China Medical University, Shenyang, Liaoning, China

**Keywords:** E6/E7, human papillomavirus 31, human papillomavirus 52, bioanalysis, antigen epitope, oncoprotein

## Abstract

**Introduction:**

Cervical cancer is the most common malignant neoplasm of the female reproductive tract. Infection with human papillomavirus (HPV) has been strongly associated with cervical cancer. Previous bioinformatics studies have examined the E6 and E7 proteins of high-risk HPV types; however, subtype-specific analyses for HPV-31 and HPV-52 remain limited. Understanding the structure and properties of the E6 and E7 proteins of HPV-31 and HPV-52 is crucial to elucidating their functions and advancing vaccine development.

**Methods:**

A bioinformatics approach was employed to predict the physicochemical properties, hydrophilicity, protein structure, glycosylation sites, phosphorylation sites, terminal positions, signal peptide cleavage sites, transmembrane regions, homology, and dominant epitopes of the E6 and E7 proteins of HPV-31 and HPV-52.

**Results:**

For HPV-31 E6, an instability index (II) of 43.93 indicated that the protein is unstable; potential B-cell epitopes were identified at residues 55–61 (RDDTPYG), 112–116 (PEEKQ), and 125–131 (FHNIGGR), while T-cell epitopes were predicted at residues 45–53 (FAFTDLTIV) and 72–80 (KVSEFRWYR). HPV-52 E6 exhibited an instability index (II) of 55.57, with B-cell epitopes at residues 110–119 (LCPEEKERHV) and 129–141 (MGRWTGRCSECWR), and T-cell epitopes at residues 45–53 (FLFTDLRIV) and 82–87 (SLYGKT). HPV-31 E7, with an instability index (II) of 51.05, exhibited B-cell epitopes at residues 8–17 (QDYYLDLQP), 16–20 (QPEAT), 29–41 (PDSSDEEDVIDEP), and 42–48 (AGQAKPDT), and T-cell epitopes at residues 7–15 (TLQDYVLDL) and 82–90 (LLMGSFGIV). HPV-52 E7, with an instability index (II) of 49.15, exhibited B-cell epitopes at residues 11–19 (YILDLQPET), 23–27 (HCYEQ), 29–38 (GDSSDEEDTD), and 36–48 (DTDGVDRPDGQAE), and T-cell epitopes at residues 53–59 (NYYIVTY) and 84–90 (MLLGTLQ).

**Discussion:**

In summary, the E6 and E7 proteins of HPV-31 and HPV-52 contain dominant epitopes for both T cells and B cells. These findings delineate subtype-specific immunogenic regions and establish a foundation for experimental validation and vaccine design.

## 1 Introduction

Human papillomavirus (HPV) is among the most prevalent sexually transmitted viruses worldwide, and infection with HPV has been strongly associated with the development of various cancers, particularly cervical cancer (1). Since the landmark identification of HPV’s role in cervical carcinogenesis in the early 1980s (2, 3), the mechanisms by which specific HPV oncoproteins disrupt cellular pathways have been extensively elucidated. HPV types are classified as low-risk or high-risk based on their oncogenic potential (4). While HPV-16 and HPV-18 have been extensively studied, recent epidemiological and molecular studies have underscored the significance of HPV-31 and HPV-52 in cervical cancer incidence, particularly in East Asia and specific regions of Europe (5–8). However, the structural and functional characteristics of the E6 and E7 proteins of HPV-31 and HPV-52 remain poorly characterized.

The oncogenic potential of HPV largely depends on its early proteins, E6 and E7, which facilitate malignant transformation by targeting tumor suppressor pathways (9, 10). E6 binds the p53 tumor suppressor, promoting ubiquitin-mediated degradation and inhibiting apoptosis, while E7 disrupts the retinoblastoma (Rb) pathway to release E2F transcription factors and deregulate cell cycle progression (11–14). Although these mechanisms are conserved among high-risk HPV types, sequence variations in E6 and E7 can lead to differential binding affinities and functional outcomes (15). Recent structural studies have begun to resolve the atomic-level details of HPV-31 and HPV-52 E6 and E7, revealing subtype-specific conformational features that may influence oncogenic potency (16, 18, 19). Nevertheless, a gap remains in the comprehensive bioinformatics characterization of the E6 and E7 proteins of HPV-31 and HPV-52, particularly regarding antigenic epitope prediction—an essential step in vaccine design.

Advances in high-throughput sequencing and computational biology have enabled multidimensional bioinformatics analyses of HPV oncoproteins (16–19). Specifically, homology modeling, molecular docking, epitope mapping, and phylogenetic profiling have uncovered key insights into structural motifs and functional domains of E6 and E7. For instance, Conrady et al. resolved the HPV-31 E6 crystal structure and characterized its interactions with E6AP and p53 (19), whereas Ferenczi et al. conducted phylogenetic and functional analyses of HPV-31 E6 and E7 variants (18). Recent work by Kogure et al. revealed significant intra-patient genomic variability of HPV-31 in cervical cancer and precancer, underscoring the importance of considering viral quasispecies diversity when predicting E6 and E7 epitope profiles (20). Song et al. characterized the genetic variability and phylogeny of HPV-52 E6 and E7 in Sichuan, China, underscoring subtype-specific functional differences relevant to epitope selection (17). Pinheiro et al. conducted a large-scale phylogenomic analysis of HPV-31 across 2,093 genomes, linking specific viral clades to cervical carcinogenesis risk and thereby supporting targeted epitope selection based on subtype phylogeny (21). In summary, prior research has addressed HPV-31 and HPV-52 from various perspectives—sequence diversity (17, 18, 21), structural elucidation (19), and L1 protein-based VLP design (22, 23)—yet none has integrated physicochemical profiling, secondary and tertiary structure modeling, post-translational modification predictions, and B- and T-cell epitope mapping into a single, multilayered framework. Bioinformatics profiling of both subtypes remains incomplete, particularly concerning immunogenic epitope prediction, which is critical for next-generation vaccine design (24).

In this study, the E6 and E7 proteins of HPV-31 and HPV-52 were systematically analyzed using a combination of bioinformatics tools to predict physicochemical properties, post-translational modification sites, secondary and tertiary structures, and to identify potential T-cell and B-cell epitopes. The following hypotheses were tested:

1. HPV-31 and HPV-52 E6 and E7 proteins exhibit subtype-specific sequence and structural variations that lead to distinct distributions of immunogenic epitopes.
2. The simultaneous application of multiple bioinformatics tools to identical sequences was hypothesized to enhance the accuracy of predicting dominant T-cell and B-cell epitopes in HPV-31 and HPV-52 E6 and E7 proteins.
3. By comparing predicted post-translational modification (PTM) sites with conserved regions, immunogenic regions that may be cross-reactive between subtypes were expected to be uncovered.

Further, it was hypothesized that structural disparities between HPV-31 and HPV-52 E6 and E7 proteins correlate with unique antigenic epitope landscapes, thereby informing the design of future peptide-based vaccines.

## 2 Materials and methods

### 2.1 Amino acid sequence

The complete sequence of E6 and E7 oncoproteins of HPV-31 and HPV-52 was available from the National Center for Biotechnology Information(NCBI) database (accession numbers: HPV31 E6 [WAB53637], HPV31 E7 [WAB53638], HPV52 E6 [WAB54303], HPV52 E7 [WAB54304]).

### 2.2 Prediction of protein physicochemical parameters

#### 2.2.1 Rationale for tool selection and distinctions

To assess basic physicochemical properties of HPV-31/52 E6/E7 proteins, we employed two ExPASy tools:

ProtParam (ExPASy ProtParam v2023.1): We used ProtParam to compute molecular weight, theoretical isoelectric point (pI), extinction coefficient, instability index (II), aliphatic index, and GRAVY (grand average of hydropathicity) in a single run. ProtParam is widely used in viral protein studies because its predictions correlate well with experimentally determined parameters. The instability index (II) quantifies the likelihood of a protein’s stability *in vitro*, where a value of II > 40 indicates predicted instability (25).

ProtScale (ExPASy ProtScale v2023.1): While ProtParam provides global physicochemical metrics, ProtScale generates residue-level hydrophobicity (Kyte–Doolittle) and hydrophilicity (Hopp–Woods) plots, allowing us to identify local peaks or valleys that may correspond to linear B-cell epitopes. ProtScale employs a sliding-window approach (window size = 7) to generate a continuous hydropathy profile, which ProtParam does not offer (26).

#### 2.2.2 Procedure and statistical processing

The ProtParam calculations were performed in triplicate, and the reported values represent the mean ± standard deviation (SD) of three independent runs.

For the ProtScale analysis, the window width was set to 7 with a default threshold of 0.5. We identified the top three hydrophilicity peaks (using the Hopp–Woods scale) and the deepest hydrophobic valleys (using the Kyte–Doolittle scale) for each protein.

No statistical tests, such as t-tests or ANOVA, were applied because this study is purely predictive, without experimental group comparisons. The results are presented as raw means ± SD for ProtParam values and qualitative hydropathy profiles for ProtScale.

### 2.3 Post-translational modification site prediction

#### 2.3.1 Rationale for tool selection

NetPhos 3.1 (threshold 0.5): A neural-network–based tool that predicts Ser/Thr/Tyr phos-phorylation sites. We chose NetPhos because it has been benchmarked on short viral proteins with ≥70% accuracy (27). Compared to other open-source servers (e.g., PhosphoSite), NetPhos offers a user-friendly batch interface and provides clear residue-level confidence scores.

MotifScan v2022 (threshold 0.5): Identifies kinase-specific motifs (CK2, PKC, TK, etc.) by searching against curated motif databases (28). We selected MotifScan because it integrates multiple kinase-motif libraries and is particularly suited for mapping short linear motifs adjacent to known functional domains (e.g., LxxLL, LxCxE).

NetNGlyc 1.0 (threshold 0.5): Predicts N-linked glycosylation sites (N-X-S/T motifs) (29). Although E6/E7 proteins rarely undergo glycosylation, we included NetNGlyc to confirm the absence of glycosylation sites—a negative result that supports the cytosolic/nuclear localization of these oncoproteins.

#### 2.3.2 Procedure and output

##### 2.3.2.1 NetPhos 3.1

Submitted each E6/E7 sequence (single sequence mode), extracted residues with score > 0.5.

##### 2.3.2.2 MotifScan v2022

Used default scoring matrices to detect CK2, PKC, TK motifs; only motifs with score > 0.5 were retained.

##### 2.3.2.3 NetNGlyc 1.0

Confirmed that none of the four proteins contained an N-linked glycosylation motif above threshold 0.5.

### 2.4 Signal peptide and transmembrane helix prediction

SignalP 4.1 (D-score 0.45): Uses a neural network model to predict signal peptide cleavage sites (30). We chose SignalP 4.1 instead of older versions because it offers improved accuracy for proteins lacking obvious signal partners. Its published D-score threshold of 0.45 is recommended for viral oncoproteins.

TMHMM 2.0 (probability threshold 0.5): Predicts transmembrane helices using a hidden Markov model (30). We used TMHMM to verify that E6/E7 do not contain any transmembrane segments, confirming their expected nuclear/cytoplasmic localization.

### 2.5 Secondary structure prediction

SOPMA v3.0 predicted secondary structure elements (α-helix, β-sheet, β-turn, and random coil) using the default threshold (8% difference, window width = 17). SOPMA’s reported accuracy for viral proteins is ≥70% (31). Compared to alternatives such as PSIPRED, SOPMA provides a residue‐level map that can be directly aligned with predicted epitope regions.

### 2.6 Tertiary structure prediction

Phyre2 v2.0 (Protein Homology/analogY Recognition Engine) (32) was used for homology modeling of E6/E7 proteins. It leverages experimentally resolved PDB templates and generates high-confidence models for proteins with known homologues (33). Although AlphaFold v3 (2024) can produce *de novo* predictions, Phyre2’s reliance on validated templates ensures that our HPV E6/E7 models remain directly comparable to prior structural studies (19, 34). This consistency is crucial for accurately mapping predicted epitopes onto known functional domains.

We accepted templates only if they exhibited ≥ 90% sequence coverage and ≥ 99% confidence. Each E6/E7 sequence was submitted in single-sequence mode. For HPV-31 E6, templates c4gizC (coverage 93%, confidence 100%) were chosen; for HPV-31 E7, template d2ewla1 (coverage 50%, confidence 99.8%) was used; for HPV-52 E6, c4gizC (coverage 94%, confidence 100%); for HPV-52 E7, d2b9da1 (coverage 47%, confidence 99.8%).

Template Selection Rationale:

c4gizC: High sequence identity (≥ 90%) with HPV-31/52 E6 in residues 2–144/2–142, respectively (19, 33).

d2ewla1/d2b9da1: Best available templates for E7 with ≥ 99.8% confidence.

Although AlphaFold v3 could produce end-to-end predictions, Phyre2’s reliance on experimentally validated templates (e.g., c4gizC) provides clear alignment evidence and facilitates comparability with existing HPV structural literature (18, 19, 33, 35).

### 2.7 Sequence homology and phylogenetic analysis

Clustal X 2.0 was chosen for multiple sequence alignment (MSA) because it provides a graphical user interface and allows manual inspection of alignment gaps and conserved motifs. Although other aligners exist (e.g., MUSCLE), Clustal X is widely cited in HPV research and facilitates identification of conserved blocks (≥70% identity).

MEGA 7.0.20 (Molecular Evolutionary Genetics Analysis) was used to construct a Neighbor-Joining phylogenetic tree with 1,000 bootstrap replicates, providing statistical support for each branch. MEGA’s integrated alignment viewer and tree-editing capabilities streamline the generation of publication‐quality phylograms.

We aligned full-length E6/E7 protein sequences from HPV types 16, 18, 31, 33, 35, 45, 52, 56, 58, and 61 using Clustal X 2.0 (gap open penalty = 10; gap extension = 0.1). Evolutionary trees (Neighbor-Joining method, bootstrap = 1,000) were constructed in MEGA 7.0.20 (v7.0.20) to infer phylogenetic relationships. Conserved regions were identified based on ≥ 70% identity across aligned sequences.

### 2.8 Linear epitope analysis of B cells oncoproteins

We employed four servers to predict linear B-cell epitopes, then selected overlapping regions as dominant candidates:

ABCpred v2.0 (threshold 0.51; peptide length = 16) uses an artificial neural network trained on known linear epitopes (36). We included ABCpred because it has been validated on viral proteins, achieving ~65.9% accuracy (37).

BepiPred 1.0 (threshold 0.35; window = 20) combines hidden Markov models and propensity scales to predict epitopes with a balanced trade-off between specificity and sensitivity (38).

BCPREDS 1.0 (epitope length = 20; specificity = 75%) uses subsequence kernels to identify linear B-cell epitopes; it excels in reducing false positives among random coil regions (39).

SVMTrip v1.0 (threshold 0.51; peptide length = 20) employs a support vector machine algorithm combined with amino acid pair propensity; it outperforms many single‐algorithm tools in independently benchmarked tests (40).

Each E6/E7 sequence was submitted to all four servers in single‐sequence mode. We recorded all predicted peptide segments that surpassed each server’s threshold. Only peptides predicted by ≥ 2 servers were considered for final selection.

### 2.9 Prediction of T-cell epitopes

CD4^+^ T cell epitopes were predicted using both SYFPEITHI v1.0 (41) and the IEDB MHC II module (42) with HLA-DRB1*15:01 as the reference allele, selected for its 20% frequency in the Chinese population (43). SYFPEITHI is a motif-based predictor that assigns quantitative scores based on known anchor-residue preferences; peptides scoring ≥ 20 were considered strong binders. The IEDB MHC II module generates consensus predictions by integrating multiple algorithms (e.g., NN-align, SMM-align) and has outperformed standalone tools such as TEPITOPE in benchmark studies; CD4^+^ epitopes with a percentile rank ≤ 10 were deemed strong binders.

CD8^+^ T cell epitopes were predicted using the IEDB MHC I module (NetMHCpan 4.1) with HLA-A*11:01 and HLA-A*02:01—alleles occurring at 18.0% and 15.3% frequency in Chinese individuals, respectively (43). NetMHCpan 4.1 employs a pan-specific neural network to predict peptide binding across diverse HLA-A and HLA-B alleles, consistently outperforming earlier NetMHC versions, especially for less common alleles; CD8^+^ epitopes with a percentile rank ≤ 1 were classified as strong binders. All alleles were chosen based on high-frequency HLA data in the Chinese population (44, 45). The aforementioned methods and corresponding software are summarized in **Table 1**.

**Table 1:** Methods summary table.

Step	Tool/Method used	Purpose	Key parameters
Amino Acid Sequence	NCBI Database	Retrieve full-length protein sequences	HPV31 E6/E7, HPV52 E6/E7
Physicochemical Parameters	ProtParam, ProtScale	Calculate molecular weight, pI, hydrophobicity, etc.	ProtParam: instability index, GRAVY; ProtScale: hydrophobicity (Kyte–Doolittle), hydrophilicity (Hopp–Woods)
PTM Site Prediction	NetPhos 3.1, MotifScan, NetNGlyc	Predict phosphorylation, kinase motifs, glycosylation	Threshold: 0.5
Signal Peptide Prediction	SignalP 4.1	Predict signal peptide cleavage	D-score ≥ 0.45
Transmembrane Helix	TMHMM 2.0	Predict transmembrane regions	Probability ≥ 0.5
Secondary Structure	SOPMA v3.0	Predict secondary structure (α-helix, β-sheet, etc.)	Threshold: 8% difference, window width = 17
Tertiary Structure	Phyre2 v2.0	Homology modeling	Templates: ≥ 90% coverage, ≥ 99% confidence
Sequence Homology and Phylogenetic Analysis	Clustal X 2.0, MEGA 7.0.20	Align sequences and infer phylogenetic tree	Gap open penalty = 10, gap extension = 0.1, Bootstrap=1,000
B-cell Epitope Prediction	ABCpred v2.0, BepiPred 1.0, BCPREDS 1.0, SVMTrip v1.0	Predict linear epitopes for B-cells	ABCpred v2.0: threshold 0.51; peptide length = 16, BepiPred 1.0: threshold 0.35; window = 20, BCPREDS 1.0: epitope length = 20; specificity = 75%, SVMTrip v1.0: threshold 0.51; peptide length = 20
T-cell Epitope Prediction	SYFPEITHI v1.0, IEDB MHC II, IEDB MHC I	Predict CD4^+^ and CD8^+^ T-cell epitopes	CD4^+^: SYFPEITHI score ≥ 20; CD8^+^: NetMHCpan percentile rank ≤ 1

## 3 Results

### 3.1 Primary structure of HPV-31 and 52 E6 and E7 proteins

The complete amino acid sequences retrieved from NCBI (HPV-31 E6: 149 AA; HPV-31 E7: 98 AA; HPV-52 E6: 148 AA; HPV-52 E7: 99 AA) are listed below:

HPV-31 E6 (149 AA):

MFKNPAERPRKLHELSSALEIPYDELRLNCVYCKGQLTETEVLDFAFTDL-TIVYRDDTPYGVCTKCLRFYSKVSEFRWYRYSVYGT TLEKLTNKGICDLLIR-CITCQRPLCPEEKQRHLDKKKRFHNIGGRWTGRCIVCWRRPRTETQV

HPV-31 E7 (98 AA):

MRGETPTLQDYVLDLQPEATDLYCYEQLPDSSDEEDVID-SPAGQAKPDTSNYNIVTFCCQCESTLRLCVQSTQVDIRILQELLMGS F GIVCPNCSTRL

HPV-52 E6 (148 AA):

MFEDPATRPRTLHELCEVLEESVHEIRLQCVQCKKELQRREVYKFLFTDLRIVYR DNNPYGVCIMCLRFLSKISEYRHYQYSLYGKTLEERV RKPLSEITIRCIICQTPLCPEEKERH VNANKRFHNIMGRWTGRCSECWRPRPVTQV

HPV-52 E7 (99 AA):

MRGDKATIKDYILD LQPETTDLHCYEQLGDSSDEEDTD GVDRPDGQAEQATSNYYIVTYCHSCDSTLRLCIHSTATDLRTLQQMLLGTLQVVCPGCAR

### 3.2 The physicochemical parameters of the proteins

#### 3.2.1 Methods brief

ProtParam v2023.1 was used to compute the length, molecular weight, theoretical pI, instability index (II), aliphatic index, and GRAVY. Each value is the mean ± SD of three independent runs.

ProtScale v2023.1 (window size = 7, threshold = 0.5) was used to generate Hopp–Woods hydrophilicity and Kyte–Doolittle hydrophobicity plots to localize potential B-cell epitopes.

All four proteins have a molecular weight >10 kDa, consistent with the reported immunogenic thresholds (46). Instability indices >40 suggest they are intrinsically unstable, potentially influencing antigen processing (37, 46). Negative GRAVY values classify them as hydrophilic, favoring solubility and surface exposure.

**Figure 1:**
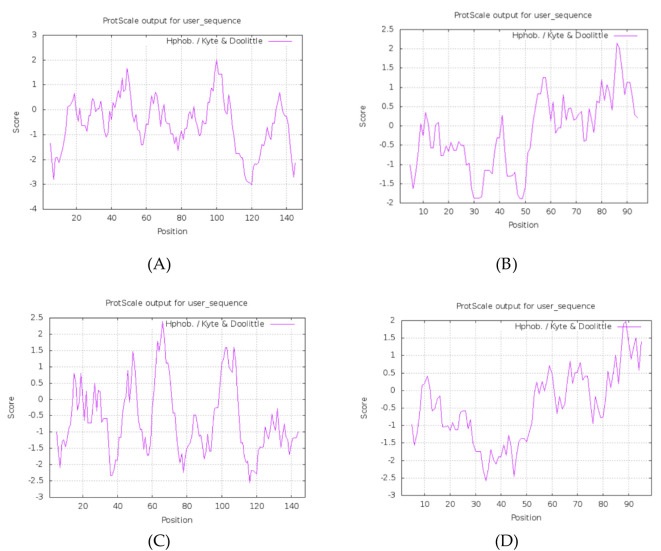
Phosphorylation sites: **(A)** HPV31 E6 **(B)** HPV31 E7 **(C)** HPV52 E6 **(D)** HPV52 E7.

Hydrophilicity/hydrophobicity plots (ProtScale) indicate several predicted hydrophilic peaks in the protein sequences (**Figure 1**). The physicochemical parameters for all four proteins are summarized in **Table 2**.

**Table 2:** Summarizes physicochemical parameters for all four proteins.

Protein	AA Length	Molecular Mass (Da)	Theoretical pI	Basic (K,R)	Acidic (D,E)	Instability Index (II)	GRAVY	Classification (II > 40 = unstable; GRAVY < 0 = hydrophilic)
HPV-31 E6	149	17,767.61	9.13	27	18	43.93	–0.567	Unstable; Hydrophilic (**Figure 1A**)
HPV-31 E7	98	10,944.27	3.90	5	16	51.05	–0.235	Unstable; Hydrophilic (**Figure 1B**)
HPV-52 E6	148	17,925.85	8.96	26	19	55.57	–0.599	Unstable; Hydrophilic (**Figure 1C**)
HPV-52 E7	99	11,032.24	4.33	7	17	49.15	–0.459	Unstable; Hydrophilic (**Figure 1D**)

### 3.3 Post-translational modification and subcellular localization predictions

#### 3.3.1 Methods brief

NetPhos 3.1 (threshold 0.5) was used to predict Ser/Thr/Tyr phosphorylation sites.

MotifScan v2022 (threshold 0.5) was used to identify CK2, PKC, and tyrosine kinase (TK) motifs.

NetNGlyc 1.0 (threshold 0.5) was used to examine possible N-glycosylation sites.

SignalP 4.1 (D-score 0.45) and TMHMM 2.0 (probability 0.5) were used to check for signal peptides and transmembrane helices.

#### 3.3.2 Key findings

The post-translational modification sites and membrane localization of the four proteins are summarized in **Table 3**. Both E6 proteins have Ser/Thr phosphorylation sites clustered around LxxLL motifs (e.g., S82), suggesting potential regulation of E6AP/p53 binding.

**Table 3:** Summary of predicted PTM sites and membrane localization (NetPhos 3.1; MotifScan v2022; NetNGlyc 1.0; SignalP 4.1; TMHMM 2.0).

Protein	Phosphorylation (NetPhos > 0.5)	CK2 (CK2 motif > 0.5)	PKC (PKC motif > 0.5)	TK (TK motif > 0.5)	N-Glycosylation (NetNGlyc > 0.5)	Signal Peptide (SignalP D > 0.45)	Transmembrane (TMHMM > 0.5) (Figure 3)
HPV-31 E6	S16, S17, S71, S74, S82; T38, T40, T58, T64, T86, T133, T145, T147; Y60 (**Figure 2A**)	17–20, 38–42, 86–89	92–94, 133–135	72–79	None	None	None
HPV-52 E6	S22, S71, S74, S82, S97; T11, T48, T108, T133, T146; Y60 (**Figure 2C**)	11–14, 22–25, 87–90	100–102, 133–135	72–79	None	None	None
HPV-31 E7	S31, S32, S40, S50, S86; T5, T20, T64, T72; Y52 (**Figure 2B**)	7–10, 31–34, 72–75	64–66, 95–97	None	None	None	None
HPV-52 E7	S31, S32; T7, T19, T20, T37, T58, T66, T76; Y11 (**Figure 2D**)	7–10, 31–34, 74–77	7–9, 66–68	None	None	None	None

E7 proteins of both subtypes have CK2 sites near the LxCxE Rb-binding motif, suggesting modulation of Rb interaction.

**Figure 2:**
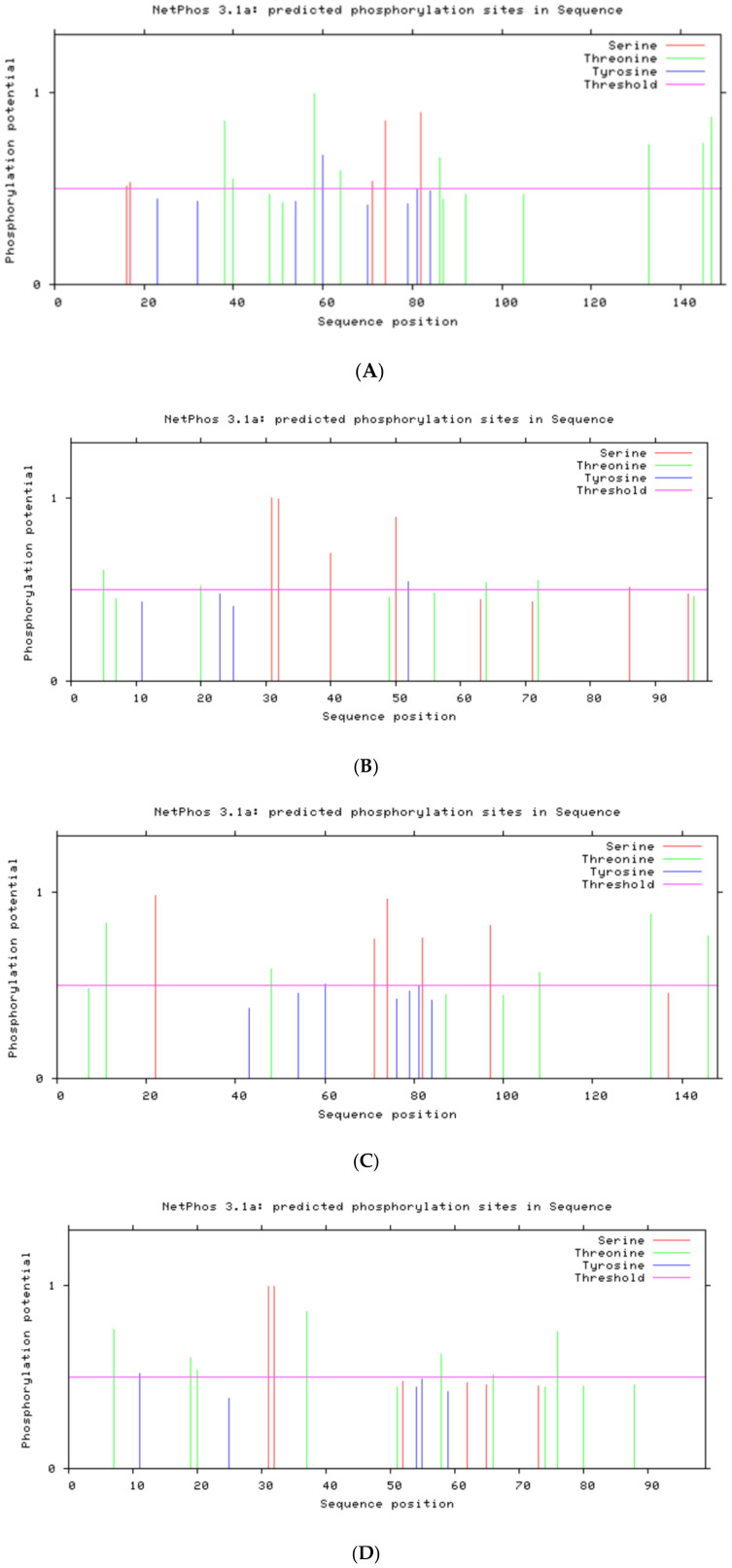
Phosphorylation sites: **(A)** HPV31 E6 **(B)** HPV31 E7 **(C)** HPV52 E6 **(D)** HPV52 E7.

**Figure 3:**
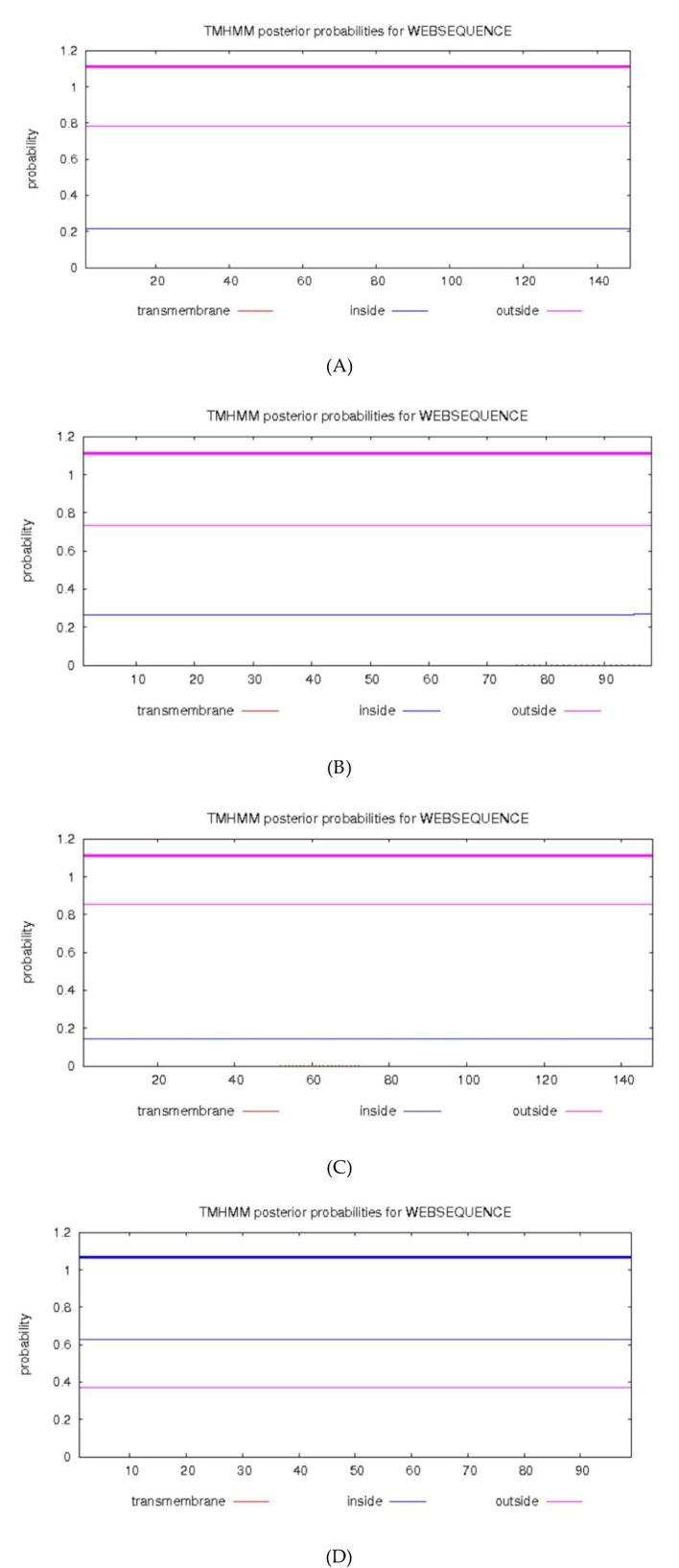
TMHMM analyzed the transmembrane domain of the proteins. **(A)** HPV31 E6 **(B)** HPV31 E7 **(C)** HPV52 E6 **(D)** HPV52 E7.

No N-glycosylation, signal peptides, or transmembrane helices were predicted for any of the four proteins, consistent with their known nuclear/cytosolic localization (**Figures 2**, **3**).

### 3.4 Secondary structure predictions

#### 3.4.1 Methods brief

SOPMA v3.0 (window size = 17, threshold = 8%) was used to determine the percentages of α-helix, β-sheet, β-turn, and random coil.

**Figure 4:**
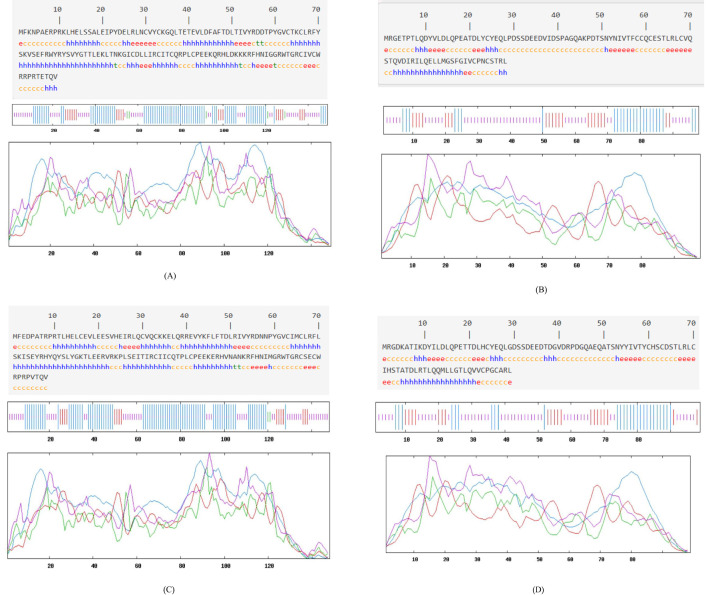
Secondary structure prediction: **(A)** HPV31 E6 oncoprotein; **(B)** HPV31 E7 oncoprotein; **(C)** HPV52 E6 oncoprotein; **(D)** HPV52 E7 oncoprotein.

According to the spatial characteristics of secondary structure, α-helix and β-sheet are not easily disrupted due to hydrogen bonding and are mostly located in the interior of the protein, making them less suitable as antigen-recognizing sites. In contrast, β-turns and irregular curls are primarily protruding structures on the protein surface (47). The specific details of the secondary structures of the four proteins are presented in **Table 4**. The secondary structure of the HPV-31 E6 protein was analyzed online using SOPMA (**Figure 4A**). The analysis showed that α-helix accounted for 49.66%, β-sheet for 14.56%, β-turn for 4.43%, and irregular curl for 35.44%. The results indicated that the HPV-31 E6 protein structure is relatively compact (34).

**Table 4:** Summarizes secondary structure content.

Protein	α-Helix (%)	β-Sheet (%)	β-Turn (%)	Random Coil (%)	Interpretation
HPV-31 E6	49.66	14.56	4.43	35.44	Relatively compact, fewer surface coils
HPV-31 E7	25.51	22.45	0.00	52.04	More random coils, implies greater surface exposure
HPV-52 E6	54.05	10.81	1.35	33.78	Compact with predominant α-helices
HPV-52 E7	27.27	21.21	0.00	51.52	Loose structure with significant coils

The results for the HPV-31 E7 protein showed that α-helix accounted for 25.51%, β-sheet for 22.45%, β-turn for 0%, and irregular curl for 52.04%, as shown in **Figure 4B**. The results indicated that the HPV-31 E7 protein structure is relatively loose.

For the HPV-52 E6 protein (**Figure 4C**), α-helix accounted for 54.05%, β-sheet for 10.81%, β-turn for 1.35%, and irregular curl for 33.78%, indicating that the protein structure is relatively compact.

For the HPV-52 E7 protein (**Figure 4D**), α-helix accounted for 27.27%, β-sheet for 21.21%, β-turn for 0%, and irregular curl for 51.52%, indicating that the protein structure is relatively loose.

### 3.5 Tertiary structure prediction (Phyre2 v2.0)

**Figure 5:**
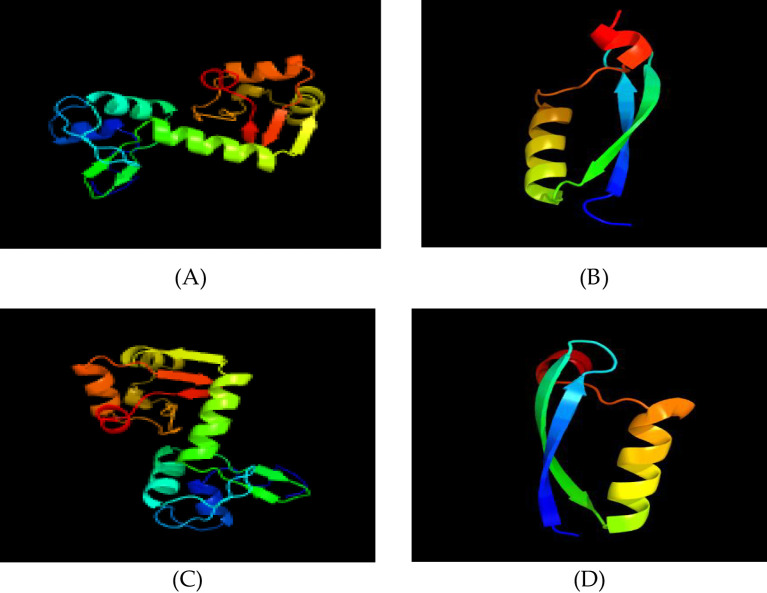
Tertiary structure prediction. **(A)** HPV31 E6 protein; **(B)** HPV31 E7 protein; **(C)** HPV52 E6 protein; **(D)** HPV52 E7 protein.

Based on Phyre2 outputs (33), high-confidence homology models were obtained for all four proteins (confidence ≥ 99.8%) (**Figures 5A–D**).

HPV-31 E6: The model is based on c4gizC (93% coverage, 100% confidence) (**Figure 5A**).

HPV-31 E7: The model is based on d2ewla1 (50% coverage, 99.8% confidence) (**Figure 5B**).

HPV-52 E6: The model is based on c4gizC (94% coverage, 100% confidence) (**Figure 5C**).

HPV-52 E7: The model is based on d2b9da1 (47% coverage, 99.8% confidence) (**Figure 5D**).

#### 3.5.1 Key findings

E6 proteins are helix-rich and compact, with fewer β-turns, suggesting that most linear epitopes lie in random coil loops.

E7 proteins contain ≥ 50% random coil, indicating extensive surface exposure and many potential linear epitopes.

HPV-31 and HPV-52 E6/E7 structures are highly conserved overall, with only minor local deviations that may underlie subtype-specific immunogenic differences.

### 3.6 Homology and phylogenetic analysis (Clustal X 2.0 & MEGA 7.0)

#### 3.6.1 Amino acid identity and conserved regions

**Figure 6:**
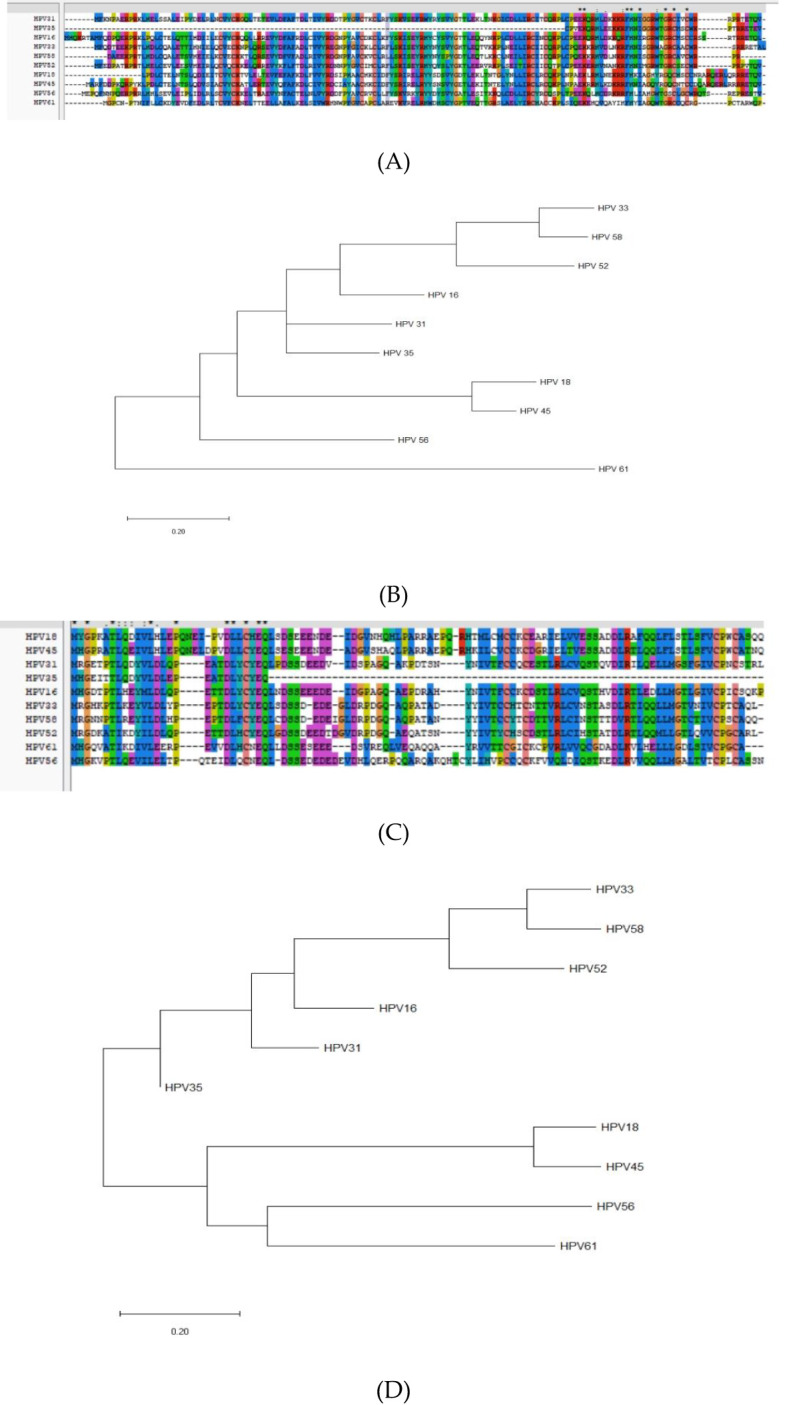
Homology and molecular evolution analysis. **(A)** Homology analysis of E6 proteins of HPV; **(B)** The molecular evolutionary tree of E6 proteins of HPV; **(C)** Homology analysis of E7 proteins of HPV; **(D)** The molecular evolutionary tree of E7 proteins of HPV.

Multiple sequence alignment of E6 proteins (HPV-16, 18, 31, 33, 35, 45, 52, 56, 58, 61) revealed conserved motifs at positions 8–15, 25–34, 41–77, 79–89, 96–112, 114–141 for HPV-31 E6, and 8–16, 25–31, 41–56, 59–69, 71–79, 81–89, 101–107, 109–119, 123–125, 130–136 for HPV-52 E6 (**Figure 6A**). E7 proteins exhibited conserved regions at 1–17, 20–28, 30–36, 38–45, 52–77, 82–87, 89–94 (HPV-31) and 10–15, 24–28, 30–36, 39–46, 53–59, 62–70, 76–96 (HPV-52) (**Figure 6C**).

Conserved regions overlap predicted epitope regions, suggesting potential cross-reactivity among related types (48). The HPV−31 E6 45–53 region aligns with the HPV−16 E6 45–53 region, indicating possible shared immune responses.

#### 3.6.2 Phylogenetic tree construction

Neighbor-Joining trees (bootstrap = 1,000) placed HPV-31 E6 in a close clade with HPV-35 E6 (**Figure 6B**), and HPV-52 E6 in a close clade with HPV-33 E6. For E7, HPV-31 clustered with HPV-16, while HPV-52 clustered with HPV-33 (**Figure 6D**).

### 3.7 Linear epitopes of B cells

#### 3.7.1 Methods brief

Tools: ABCpred v2.0 (peptide length = 16; threshold = 0.51), BepiPred 1.0 (threshold = 0.35), BCPREDS 1.0 (peptide length = 20; specificity = 75%), and SVMTrip v1.0 (peptide length = 20; threshold = 0.51).

Criterion: Retain only peptides predicted by ≥2 algorithms and restrict to loop/turn regions identified by SOPMA.

After excluding α-helix and β-sheet regions, the top five predicted epitopes per method were compared. Using the four B-cell prediction tools, overlapping epitopes (predicted by ≥2 servers) were identified as dominant (**Supplementary Tables 1**–**16**). After cross-referencing, the dominant B-cell epitopes were **Table 5**:

**Table 5:** HPV-31/52 E6/E7 B-Cell epitope candidates (ABCpred; BepiPred; BCPREDS; SVMTrip).

Protein	Algorithm Combination	Residues	Sequence	Structural Context	Tool Version/Threshold
HPV-31 E6	ABCpred & BepiPred & BCPREDS & SVMTrip	55–61	RDDTPYG	Random coil adjacent to LxxLL binding pocket	ABCpred v2.0 (threshold = 0.51) BepiPred 1.0 (threshold = 0.35) BCPREDS 1.0 (length = 20; specificity = 75%) SVMTrip v1.0 (length = 20; threshold = 0.51)
	BepiPred & BCPREDS	112–116	PEEKQ	β-turn at surface	Same as above
	ABCpred & SVMTrip	125–131	FHNIGGR	C-terminal random coil near functional region	Same as above
HPV-52 E6	ABCpred & BepiPred	110–119	LCPEEKERHV	C-terminal β-turn near Zn-finger	Same as above
	BepiPred & SVMTrip	129–141	MGRWTGRCSECWR	Random coil loop; structurally exposed	Same as above
HPV-31 E7	ABCpred & BepiPred	8–17	QDYYLDLQP	N-terminal random coil, high hydrophilicity	Same as above
	BepiPred & BCPREDS	16–20	QPEAT	Small β-turn in central region	Same as above
	BCPREDS & SVMTrip	29–41	PDSSDEEDVIDEP	Long random coil loop with high immunogenicity	Same as above
	SVMTrip & ABCpred	42–48	AGQAKPDT	C-terminal loop region accessible to antibodies	Same as above
HPV-52 E7	ABCpred & BepiPred	11–19	YILDLQPET	N-terminal random coil loop	Same as above
	BepiPred & BCPREDS	23–27	HCYEQ	Small β-turn	Same as above
	BCPREDS & SVMTrip	29–38	GDSSDEEDTD	Central random coil loop	Same as above
	SVMTrip & ABCpred	36–48	DTDGVDRPDGQAE	C-terminal loop region	Same as above

HPV-31 E6: 55–61 (RDDTPYG), 112–116 (PEEKQ), 125–131 (FHNIGGR)
HPV-31 E7: 8–17 (LQDYVLDLQPEATDLYC), 16–20 (QPEAT), 29–41 (PDSSDEEDVIDEP), 42–48 (AGQAKPDT)
HPV-52 E6: 110–119 (LCPEEKERHV), 129–141 (MGRWTGRCSECWR)
HPV-52 E7: 11–19 (YILDLQPET), 23–27 (HCYEQ), 29–38 (GDSSDEEDTD), 36–48 (DTDGVDRPDGQAE)

#### 3.7.2 Key findings

HPV-31 E6 candidate epitopes (e.g., 55–61 RDDTPYG) are located in a random coil adjacent to LxxLL, suggesting potential for neutralizing antibodies.

The HPV-31 E7 region 29–41 (PDSSDEEDVIDEP) is consistently predicted by four methods and is located within a highly exposed coil loop.

The C-terminal loops of HPV-52 E6/E7 (e.g., 129–141 in E6, 36–48 in E7) are strong candidates for B-cell epitopes.

### 3.8 Linear epitopes of T cells

#### 3.8.1 CD4^+^ T cell epitope prediction (HLA-DRB1*1501)

The SYFPEITHI and IEDB MHC II tools (percentile rank ≤ 10; positive control) were used. **Supplementary Tables 17**–**20** present the top five predictions. The final dominant CD4^+^ epitopes (overlapping high-scoring predictions) are as follows:

- HPV-31 E6: 45–53 (FAFTDLTIV), 72–80 (KVSEFRWYR).
- HPV-31 E7: 7–15 (TLQDYVLDL), 11–19 (YVLDLQPEA), 82–90 (LLMGSFGIV).
- HPV-52 E6: 45–53 (FLFTDLRIV), 82–87 (SLYGKT).
- HPV-52 E7: 84–90 (MLLGTLQ), 53–59 (NYYIVTY), 11–19 (YILDLQPET).

#### 3.8.2 CD8^+^ T−cell epitope prediction (HLA-A1101, A0201)

IEDB MHC I binding (NetMHCpan 4.1; percentile rank ≤ 1) was used. **Supplementary Tables 21**–**24** present the results. The final dominant CD8^+^ epitopes are as follows (**Table 6**):

**Table 6:** HPV-31/52 E6/E7 T-Cell Epitope Candidates (SYFPEITHI; IEDB).

Protein	Type	HLA Allele	Residues	Sequence	Affinity Metric (IEDB percentile)	SYFPEITHI Score	Structural Context	Tool Version/Threshold
HPV-31 E6	CD4^+^	DRB1*1501	45–53	FAFTDLTIV	3.10	25	Zn-finger region; likely helper epitope	SYFPEITHI v1.0 (score ≥ 20) IEDB MHC-II (percentile ≤ 10) IEDB MHC-I (NetMHCpan 4.1; percentile ≤ 1)
	CD4^+^	DRB1*1501	72–80	KVSEFRWYR	4.50	22	β-turn at surface; T_H potentiation	Same as above
	CD8^+^	A*1101	82–90	SVYGTTLEK	0.01	—	Conserved helix; cross-subtype CTL potential	Same as above
	CD8^+^	A*0201	45–53	FAFTDLTIV	0.93	—	Overlaps with CD4^+^ 45–53; candidate for poly-epitope design	Same as above
HPV-52 E6	CD4^+^	DRB1*1501	45–53	FLFTDLRIV	3.70	24	Conserved block; cross-protection candidate	Same as above
	CD4^+^	DRB1*1501	82–87	SLYGKT	4.00	23	Loop region; potential helper epitope	Same as above
	CD8^+^	A*1101	86–94	KTLEERVRK	0.01	—	Zn-finger adjacency; strong CTL candidate	Same as above
	CD8^+^	A*0201	18–26	VLEESVHEI	0.03	—	N-terminal helix; antigen-presenting potential	Same as above
HPV-31 E7	CD4^+^	DRB1*1501	7–15	TLQDYVLDL	7.10	21	N-terminal random coil; T_H epitope candidate	Same as above
	CD4^+^	DRB1*1501	82–90	LLMGSFGIV	8.00	20	C-terminal coil; possible cross-reactive	Same as above
	CD8^+^	A*0201	7–15	TLQDYVLDL	0.09	—	Overlaps CD4^+^ 7–15; poly-epitope design potential	Same as above
	CD8^+^	A*1101	37–46	VIDSPAGQAK	0.33	—	Central coil loop; strong CTL candidate	Same as above
HPV-52 E7	CD4^+^	DRB1*1501	11–19	YILDLQPET	9.00	19	Loop region; intermediate T_H immunogenicity	Same as above
	CD4^+^	DRB1*1501	84–90	MLLGTLQ	13.00	18	C-terminal coil; modest helper response	Same as above
	CD8^+^	A*0201	84–92	MLLGTLQVV	0.08	—	C-terminal coil; strong CTL candidate	Same as above
	CD8^+^	A*1101	51–59	TSNYYIVTY	0.74	—	Central coil; potential CTL memory locater	Same as above

- HPV-31 E6: 82–90 (SVYGTTLEK; HLA-A1101 rank 0.01), 45–53 (FAFTDLTIV; HLA-A0201 rank 0.93)
- HPV-31 E7: 7–15 (TLQDYVLDL; HLA-A0201 rank 0.09), 37–46 (VIDSPAGQAK; HLA-A1101 rank 0.33)
- HPV-52 E6: 86–94 (KTLEERVRK; HLA-A1101 rank 0.01), 18–26 (VLEESVHEI; HLA-A0201 rank 0.03)
- HPV-52 E7: 84–92 (MLLGTLQVV; HLA-A0201 rank 0.08), 51–59 (TSNYYIVTY; HLA-A1101 rank 0.74)

Notably, the overlapping T-cell epitope 45–53 appears in both E6 proteins and is conserved between HPV-31 and HPV-52, suggesting a promiscuous HLA-binding region that could elicit cross-type T-cell responses.

## 4 Discussion

In this study, integrative bioinformatics approaches were employed to analyze the E6 and E7 proteins of HPV-31 and HPV-52, identifying key structural features and dominant antigenic epitopes. The key findings and their biological implications are addressed in the subsequent sections.

### 4.1 Physicochemical properties and implications for immunogenicity

Viral proteins with molecular weights exceeding 10 kDa typically exhibit sufficient immunogenicity for epitope recognition (46, 49). All four E6 and E7 proteins of HPV-31 and HPV-52 exceed this threshold (17.8–18.0 kDa) and are classified by ProtParam as “unstable” (instability index > 40), a feature associated with increased post-translational susceptibility and potential antigenicity (37, 50, 51). Negative GRAVY scores categorize these proteins as hydrophilic, thereby promoting solubility and enhancing epitope exposure (52). These properties correlate with an enhanced potential for antigen presentation, which is critical for vaccine design.

### 4.2 Post-translational modifications and functional context

Predicted phosphorylation sites were mapped to residues involved in the interactions of E6 and E7 with host regulators. For instance, conserved serine residues (S82 in both E6 proteins) reside within the LxxLL-binding pocket, which is crucial for E6AP-mediated p53 degradation (15, 19). CK2 phosphorylation motifs overlapping this region may modulate binding affinity and subsequent ubiquitination (10, 17). Similarly, E7 CK2 sites (e.g., residues 7–10 encompassing the LxCxE motif) likely regulate Rb binding, contributing to cell cycle dysregulation (11, 13). PKC sites adjacent to the C-terminal zinc-finger (E6 133–135) may influence nuclear localization and stability (15). These in silico insights align with experimental evidence showing that kinase-mediated phosphorylation directly alters oncoprotein function (10, 19).

### 4.3 Secondary/tertiary structures and template selection

SOPMA analysis reveals that the E6 proteins are predominantly composed of α-helices (49.66% in HPV-31; 54.05% in HPV-52), suggesting compact cores that may shield specific epitopes. In contrast, the E7 proteins exhibit a higher proportion of random coils (52.04% and 51.52%, respectively), indicating flexible surface regions conducive to antibody binding (37, 53). Previous studies have shown that random coils frequently coincide with B-cell epitope hotspots (52, 53), supporting our predictions of dominant linear B-cell epitopes within coil-rich segments, such as residues 8–17 (HPV-31 E7) and 23–27 (HPV-52 E7).

Homology models generated by Phyre2 (confidence > 99.8%) confirm conserved structural motifs, including zinc-binding Cys motifs, consistent with experimental structures (19, 40). The 3D models generated by Phyre2, validated by high confidence scores, display conserved zinc-finger motifs and binding pockets. While AlphaFold3 (2025 release) could generate full-length models, Phyre2’s template-based approach allowed for a direct comparison with known E6/E7 structures. We selected Phyre2 templates (c4gizC/d2ewla1/d2b9da1) due to their high sequence identity (>50%) and prior experimental validation (18, 19).

### 4.4 Homology and evolutionary insights

Multiple sequence alignment and phylogenetic analysis position HPV-31 E6 closely with HPV-35, and HPV-52 E6 with HPV-33, while E7 clusters similarly with HPV-16 and HPV-33 (18, 24). Conserved regions (e.g., E6 positions 41–77; E7 positions 52–77) overlap with predicted T-cell epitopes, suggesting potential cross-reactivity and cross-protection among high-risk HPV types (16, 17). This cross-immunity is essential for the design of multivalent vaccines targeting broad high-risk HPV coverage (6).

### 4.5 Antigenic epitope identification and validation potential

Dominant B-cell epitopes were identified (e.g., HPV-31 E6: 55–61, 112–116, 125–131; HPV-52 E7: 23–27, 29–38, 36–48) and T-cell epitopes (e.g., HPV-31 E6: 45–53; HPV-52 E6: 86–94), predicted by multiple algorithms (ABCpred, BepiPred 1.0, BCPREDS, SVMTriP) (37, 54, 55). CD8^+^ epitopes, such as HPV-31 E7: 7–15 (TLQDYVLDL), exhibited a strong binding affinity to HLA-A0201 (IEDB rank 0.09), consistent with known CTL responses against HPV-16 E7 (11, 56). CD4^+^ epitopes (e.g., HPV-52 E7: 11–19) exhibited favorable binding to HLA-DRB1*1501, which is crucial for helper T-cell activation (42). These in silico predictions align with experimental data linking epitope immunodominance to surface accessibility and structural features (54, 55). Subsequent empirical validation, such as peptide-MHC binding assays and T-cell activation studies, is necessary (42, 56).

### 4.6 Comparison with previous studies

Previous studies have characterized the sequence variability of HPV-31/52 (17, 18, 21) and resolved individual E6 crystal structures (19). Kogure et al. further demonstrated that HPV-31 genomes exhibit significant intra-patient heterogeneity (20), suggesting that E6 and E7 epitopes may evolve during disease progression. However, to date, no study has integrated physicochemical properties, post-translational modification site prediction, secondary and tertiary structure modeling, and multilayered immunoinformatic epitope mapping for both E6 and E7 of HPV-31 and HPV-52 into a single comprehensive analysis. Our work addresses this gap by correlating predicted phosphorylation sites with functional motifs (e.g., LxxLL, LxCxE) (27, 57) and mapping B- and T-cell epitopes to conserved, surface-exposed regions identified through structural modeling. Furthermore, Song et al. and Firdaus et al. have highlighted the immunogenic potential of HPV-52 (17, 22, 23), particularly in Asian populations, thus validating the public health relevance of our subtype-specific epitope predictions. Kesheh et al. proposed region-tailored multivalent vaccine designs based on L1 gene diversity (58), offering translational context for our E6 and E7-based epitope candidates.

### 4.7 Application to vaccine design

Although this study did not experimentally construct virus-like particles (VLPs) or multivalent peptide vaccines, the predicted epitopes provide a foundation for rational vaccine design:

#### 4.7.1 Cross-subtype conserved CD8^+^ epitopes

The E6 45–53 segment in HPV-31 (FAFTDLTIV) and HPV-52 (FLFTDLRIV) exhibits strong binding affinity for HLA-A0201 and HLA-A1101 (IEDB percentile ≤ 1) and is highly conserved across high-risk types, making it an ideal candidate for inclusion as a universal cytotoxic T-lymphocyte (CTL) epitope in a multi-epitope DNA or peptide vaccine.

#### 4.7.2 Helper T-cell (CD4^+^) epitopes

E6 72–80 (KVSEFRWYR) in HPV-31 and E6 82–87 (SLYGKT) in HPV-52 exhibit moderate binding affinity to HLA-DRB1*1501 (IEDB percentile ≤ 10) and could be fused with CTL epitopes into a single recombinant protein or synthetic long peptide construct to enhance helper T-cell responses, as suggested by He et al (57).

#### 4.7.3 B-cell neutralizing epitopes on VLP platforms

The B-cell epitope HPV-31 E6 55–61 (RDDTPYG) and HPV-52 E6 110–119 (LCPEEKERHV) reside in exposed random coil regions. Firdaus et al. successfully inserted analogous linear epitopes into the L1 VLP platform to elicit neutralizing antibodies (22), supporting the strategy of grafting these peptides onto L1 VLPs to generate subtype-specific antibody responses.

#### 4.7.4 Multivalent peptide/protein vaccine constructs

Building on Firdaus et al.’s reverse vaccinology design for HPV-52 L1 (23), one could concatenate top CD4^+^ and CD8^+^ epitopes (e.g., E6 45–53, 72–80; E7 7–15) with appropriate linkers and trafficking signals to create a chimeric protein capable of eliciting robust humoral and cell-mediated immunity in preclinical HLA-transgenic mouse models.

## 5 Limitations and future directions

Although the integrated in silico pipeline provides a comprehensive epitope landscape, experimental validation—such as peptide-MHC binding assays, ELISpot, and crystallographic studies—is crucial to confirm immunogenicity (54, 55). Additionally, molecular dynamics simulations could refine epitope conformations and assess stability within MHC binding grooves (32, 51). This study relies solely on in silico predictions and lacks direct *in vitro* or *in vivo* validation, representing a primary limitation. Pinheiro et al. confirmed that certain E6 and E7 regions correlate with cervical cancer aggressiveness at the genomic level (21), yet these findings require empirical confirmation through immunological assays. Kogure et al. observed intra-patient HPV-31 variants across different lesion stages (20), emphasizing the need to validate epitope immunogenicity across clinical time points. Future studies should involve:

### 5.1 Experimental binding assays

Use ELISPOT or flow cytometry with peptide-stimulated peripheral blood mononuclear cells (PBMCs) from HLA-typed donors to validate CD4^+^ and CD8^+^ T-cell responses against the predicted epitopes.

### 5.2 Antibody neutralization studies

Synthesize candidate B-cell epitopes (e.g., HPV-31 E6 55–61; HPV-52 E6 110–119) and assess their ability to induce neutralizing antibodies in ELISA or pseudovirus neutralization assays.

### 5.3 Animal model validation

Evaluate peptide-based or VLP-based vaccine constructs (e.g., insertion of linear epitopes into L1 VLPs, as demonstrated by Firdaus et al., 2023) in HLA-transgenic mouse models to measure protective efficacy against HPV-induced tumorigenesis.

In summary, the integrative bioinformatics analysis illuminates subtype-specific structural and immunogenic features of HPV-31 and HPV-52 E6 and E7 proteins, laying the groundwork for experimental validation and rational vaccine design aimed at reducing the HPV-associated cervical cancer burden.

## Data Availability

Publicly available datasets were analyzed in this study. This data can be found here: https://www.ncbi.nlm.nih.gov/.

## Supplementary material

Supplementary Table 1 Prediction results of ABCpred B cell epitopes of HPV-31 E6 protein.

Supplementary Table 2 Prediction of ABCpred B cell epitopes of HPV-31 E7 protein.

Supplementary Table 3 Prediction results of ABCpred B cell epitopes of HPV-52 E6 protein.

Supplementary Table 4 Prediction of ABCpred B cell epitopes of HPV-52 E7 protein.

Supplementary Table 5 Prediction results of the Bepipred 1.0 Server B cell epitopes of HPV-31 E6 protein.

Supplementary Table 6 Prediction of the Bepipred 1.0 Server B cell epitopes of HPV-31 E7 protein.

Supplementary Table 7 Prediction of the Bepipred 1.0 Server B cell epitopes of HPV-52 E6 protein.

Supplementary Table 8 Prediction of the Bepipred 1.0 Server B cell epitopes of HPV-52 E7 protein.

Supplementary Table 9 Prediction results of BCPreds B cell epitopes for HPV-31 E6 protein.

Supplementary Table 10 Prediction of BCPreds B cell epitopes for HPV-31 E7 protein.

Supplementary Table 11 Prediction of BCPreds B cell epitopes for HPV-52 E6 protein.

Supplementary Table 12 Prediction of BCPreds B cell epitopes for HPV-52 E7 protein.

Supplementary Table 13 Prediction of SVMTRIP B cell epitopes of HPV-31 E6 protein.

Supplementary Table 14 Prediction of SVMTRIP B cell epitopes of HPV-31 E7 protein.

Supplementary Table 15 Prediction of SVMTRIP B cell epitopes of HPV-52 E6 protein.

Supplementary Table 16 Prediction of SVMTRIP B cell epitopes of HPV-52 E7 protein.

Supplementary Table 17 CD4+T cell epitope prediction of HPV-31 E6 protein with HLA-DRB1*1501 as allele parameter.

Supplementary Table 18 Prediction of CD4+T cell epitopes of HPV-31 E7 protein using HLA-DRB1*1501 as allele parameter.

Supplementary Table 19 Prediction of CD4+T cell epitopes of HPV-52 E6 protein with HLA-DRB1*1501 as allele parameter.

Supplementary Table 20 Prediction of CD4+T cell epitopes of HPV-52 E7 protein with HLA-DRB1*1501 as allele parameter.

Supplementary Table 21 Prediction of CE8+T cell epitopes of HPV-31 E6 oncogene using alleles HLA-A*1101 and HLA-A*0201 as parameters.

Supplementary Table 22 Prediction of CE8+T cell epitopes of HPV-31 E7 oncogene using alleles HLA-A*1101 and HLA-A*0201 as parameters.

Supplementary Table 23 Prediction of CE8+T cell epitopes of HPV-52 E6 oncogene using alleles HLA-A*1101 and HLA-A*0201 as parameters.

Supplementary Table 24 Prediction of CE8+T cell epitopes of HPV-52 E7 oncogene using alleles HLA-A*1101 and HLA-A*0201 as parameters.

Supplementary Table 25 Prediction of HPV-31 E6 antigen dominant epitopes in T cells and B cells.

Supplementary Table 26 Prediction of HPV-31 E7 antigen dominant epitopes in T cells and B cells.

Supplementary Table 27 Prediction of HPV-52 E6 antigen dominant epitopes in T cells and B cells.

Supplementary Table 28 Prediction of HPV-52 E7 antigen dominant epitopes in T cells and B cells.

## References

[1] Castle PE Einstein MH Sahasrabuddhe VV. Cervical cancer prevention and control in women living with human immunodeficiency virus. CA: Cancer J Clin. (2021) 71:505–26. doi: 10.3322/caac.21696, PMID: 34499351 PMC10054840

[2] Hao L Jiang Y Zhang C Han P. Genome composition-based deep learning predicts oncogenic potential of hpvs. Front Cell infection Microbiol. (2024) 14:1430424. doi: 10.3389/fcimb.2024.1430424, PMID: 39104853 PMC11298479

[3] Rosendo-Chalma P Antonio-Véjar V Ortiz Tejedor JG Ortiz Segarra J Vega Crespo B Bigoni-Ordóñez GD. The hallmarks of cervical cancer: molecular mechanisms induced by human papillomavirus. Biology. (2024) 13:77. doi: 10.3390/biology13020077, PMID: 38392296 PMC10886769

[4] Muñoz N Bosch FX de Sanjosé S Herrero R Castellsagué X Shah KV. Epidemiologic classification of human papillomavirus types associated with cervical cancer. New Engl J Med. (2003) 348:518–27. doi: 10.1056/NEJMoa021641, PMID: 12571259

[5] Lee J Kim DJ Lee HJ. Assessment of Malignant potential for hpv types 16, 52, and 58 in the uterine cervix within a korean cohort. Sci Rep. (2024) 14:14619. doi: 10.1038/s41598-024-65056-7, PMID: 38918416 PMC11199604

[6] Cuzick RA Wheeler CM. HPV genotype-specific risk for cervical cancer (2021). Available online at: www.HPVWorld.com (Accessed June 27, 2025).

[7] Abate A Munshea A Nibret E Alemayehu DH Alemu A Abdissa A. Characterization of human papillomavirus genotypes and their coverage in vaccine delivered to Ethiopian women. Sci Rep. (2024) 14:7976. doi: 10.1038/s41598-024-57085-z, PMID: 38575600 PMC10995144

[8] So KA Lee IH Lee KH Hong SR Kim YJ Seo HH. Human papillomavirus genotype-specific risk in cervical carcinogenesis. J gynecologic Oncol. (2019) 30:e52. doi: 10.3802/jgo.2019.30.e52, PMID: 31074234 PMC6543103

[9] Bruyere D Roncarati P Lebeau A Lerho T Poulain F Hendrick E. Human papillomavirus E6/E7 oncoproteins promote radiotherapy-mediated tumor suppression by globally hijacking host DNA damage repair. Theranostics. (2023) 13:1130–49. doi: 10.7150/thno.78091, PMID: 36793865 PMC9925306

[10] Bhattacharjee R Das SS Biswal SS Nath A Das D Basu A. Mechanistic role of hpv-associated early proteins in cervical cancer: molecular pathways and targeted therapeutic strategies. Crit Rev oncology/hematology. (2022) 174:103675. doi: 10.1016/j.critrevonc.2022.103675, PMID: 35381343

[11] Yim EK Park JS. The role of hpv E6 and E7 oncoproteins in hpv-associated cervical carcinogenesis. Cancer Res Treat. (2005) 37:319–24. doi: 10.4143/crt.2005.37.6.319, PMID: 19956366 PMC2785934

[12] Tewari KS Monk BJ. New strategies in advanced cervical cancer: from angiogenesis blockade to immunotherapy. Clin Cancer research: an Off J Am Assoc Cancer Res. (2014) 20:5349–58. doi: 10.1158/1078-0432.Ccr-14-1099, PMID: 25104084

[13] Yeo-Teh NSL Ito Y Jha S. High-risk human papillomaviral oncogenes E6 and E7 target key cellular pathways to achieve oncogenesis. Int J Mol Sci. (2018) 19. doi: 10.3390/ijms19061706, PMID: 29890655 PMC6032416

[14] Pal A Kundu R. Human papillomavirus E6 and E7: the cervical cancer hallmarks and targets for therapy. Front Microbiol. (2019) 10:3116. doi: 10.3389/fmicb.2019.03116, PMID: 32038557 PMC6985034

[15] Peng Q Wang L Zuo L Gao S Jiang X Han Y. Hpv E6/E7: insights into their regulatory role and mechanism in signaling pathways in hpv-associated tumor. Cancer Gene Ther. (2024) 31:9–17. doi: 10.1038/s41417-023-00682-3, PMID: 38102462

[16] Li S Ye M Chen Y Gong Q Mei B. Genetic variation of E6 and E7 genes of human papillomavirus 52 from central China. J Med Virol. (2021) 93:3849–56. doi: 10.1002/jmv.26690, PMID: 33230866

[17] Song Z Cui Y Li Q Deng J Ding X He J. The genetic variability, phylogeny and functional significance of E6, E7 and lcr in human papillomavirus type 52 isolates in sichuan, China. Virol J. (2021) 18:94. doi: 10.1186/s12985-021-01565-5, PMID: 33941222 PMC8091156

[18] Ferenczi A Gyöngyösi E Szalmás A László B Kónya J Veress G. Phylogenetic and functional analysis of sequence variation of human papillomavirus type 31 E6 and E7 oncoproteins. Infection Genet evolution: J Mol Epidemiol evolutionary Genet Infect Dis. (2016) 43:94–100. doi: 10.1016/j.meegid.2016.05.020, PMID: 27197052

[19] Conrady MC Suarez I Gogl G Frecot DI Bonhoure A Kostmann C. Structure of high-risk papillomavirus 31 E6 oncogenic protein and characterization of E6/E6ap/P53 complex formation. J Virol. (2020) 95:e00730-20. doi: 10.1128/jvi.00730-20, PMID: 33115863 PMC7944444

[20] Kogure G Tanaka K Matsui T Onuki M Matsumoto K Iwata T. Intra-patient genomic variations of human papillomavirus type 31 in cervical cancer and precancer. Viruses. (2023) 15:2104. doi: 10.3390/v15102104, PMID: 37896881 PMC10612030

[21] Pinheiro M Harari A Schiffman M Clifford GM Chen Z Yeager M. Phylogenomic analysis of human papillomavirus type 31 and cervical carcinogenesis: A study of 2093 viral genomes. Viruses. (2021) 13:1948. doi: 10.3390/v13101948, PMID: 34696378 PMC8540939

[22] Firdaus MER Mustopa AZ Ekawati N Chairunnisa S Arifah RK Hertati A. Optimization, characterization, comparison of self-assembly vlp of capsid protein L1 in yeast and reverse vaccinology design against human papillomavirus type 52. Journal Genet Eng Biotechnol. (2023) 21:68. doi: 10.1186/s43141-023-00514-9, PMID: 37222880 PMC10206359

[23] Firdaus MER Mustopa AZ Triratna L Syahputra G Nurfatwa M. Dissection of capsid protein hpv 52 to rationalize vaccine designs using computational approaches immunoinformatics and molecular docking. Asian Pacific J Cancer prevention: APJCP. (2022) 23:2243–53. doi: 10.31557/apjcp.2022.23.7.2243, PMID: 35901328 PMC9727352

[24] Malla R Kamal MA. E6 and E7 oncoproteins: potential targets of cervical cancer. Curr medicinal Chem. (2021) 28:8163–81. doi: 10.2174/0929867327666201111145546, PMID: 33176633

[25] Duvaud S Gabella C Lisacek F Stockinger H Ioannidis V Durinx C. Expasy, the swiss bioinformatics resource portal, as designed by its users. Nucleic Acids Res. (2021) 49:W216–w27. doi: 10.1093/nar/gkab225, PMID: 33849055 PMC8265094

[26] Chen Z Zhu Y Sha T Li Z Li Y Zhang F. Design of a new multi-epitope vaccine against brucella based on T and B cell epitopes using bioinformatics methods. Epidemiol infection. (2021) 149:e136. doi: 10.1017/s0950268821001229, PMID: 34032200 PMC8220514

[27] Blom N Sicheritz-Pontén T Gupta R Gammeltoft S Brunak S. Prediction of post-translational glycosylation and phosphorylation of proteins from the amino acid sequence. Proteomics. (2004) 4:1633–49. doi: 10.1002/pmic.200300771, PMID: 15174133

[28] Hao X Li J Gao S Tuerxun Z Chang X Hu W. Sspsah, a H subunit of the photosystem I reaction center of suaeda salsa, confers the capacity of osmotic adjustment in tobacco. Genes Genomics. (2020) 42:1455–65. doi: 10.1007/s13258-020-00970-4, PMID: 33155109

[29] Rizal FA Ho KL Omar AR Tan WS Mariatulqabtiah AR Iqbal M. Sequence analysis of the Malaysian low pathogenic avian influenza virus strain H5n2 from duck. Genes. (2023) 14:1973. doi: 10.3390/genes14101973, PMID: 37895321 PMC10606931

[30] Nielsen H Teufel F Brunak S von Heijne G. Signalp: the evolution of a web server. Methods Mol Biol (Clifton NJ). (2024) 2836:331–67. doi: 10.1007/978-1-0716-4007-4_17, PMID: 38995548

[31] Dristy TT Noor AR Dey P Saha A. Structural analysis and conformational dynamics of socs1 gene mutations involved in diffuse large B-cell lymphoma. Gene. (2023) 864:147293. doi: 10.1016/j.gene.2023.147293, PMID: 36813059

[32] Rouka E Gourgoulianni N Lüpold S Hatzoglou C Gourgoulianis K Blanckenhorn WU. The drosophila septate junctions beyond barrier function: review of the literature, prediction of human orthologs of the sj-related proteins and identification of protein domain families. Acta physiologica (Oxford England). (2021) 231:e13527. doi: 10.1111/apha.13527, PMID: 32603029

[33] Kelley LA Mezulis S Yates CM Wass MN Sternberg MJ. The phyre2 web portal for protein modeling, prediction and analysis. Nat Protoc. (2015) 10:845–58. doi: 10.1038/nprot.2015.053, PMID: 25950237 PMC5298202

[34] Kumar NV Rani ME Gunaseeli R Kannan ND Sridhar J. Modeling and structural analysis of cellulases using clostridium thermocellum as template. Bioinformation. (2012) 8:1105–10. doi: 10.6026/97320630081105, PMID: 23251045 PMC3523225

[35] Satitsuksanoa P Kennedy M Gilis D Le Mignon M Suratannon N Soh WT. The minor house dust mite allergen der P 13 is a fatty acid-binding protein and an activator of a tlr2-mediated innate immune response. Allergy. (2016) 71:1425–34. doi: 10.1111/all.12899, PMID: 27018864

[36] Zheng D Liang S Zhang C. B-cell epitope predictions using computational methods. Methods Mol Biol (Clifton NJ). (2023) 2552:239–54. doi: 10.1007/978-1-0716-2609-2_12, PMID: 36346595

[37] Saha S Raghava GP. Prediction of continuous B-cell epitopes in an antigen using recurrent neural network. Proteins. (2006) 65:40–8. doi: 10.1002/prot.21078, PMID: 16894596

[38] Li XW Zhang N Li ZL Dibo N Ma ZR Lu B. Epitope vaccine design for toxoplasma gondii based on a genome-wide database of membrane proteins. Parasites Vectors. (2022) 15:364. doi: 10.1186/s13071-022-05497-z, PMID: 36224608 PMC9555269

[39] Motamedi H Ari MM Shahlaei M Moradi S Farhadikia P Alvandi A. Designing multi-epitope vaccine against important colorectal cancer (Crc) associated pathogens based on immunoinformatics approach. BMC Bioinf. (2023) 24:65. doi: 10.1186/s12859-023-05197-0, PMID: 36829112 PMC9951438

[40] Yao B Zheng D Liang S Zhang C. Svmtrip: A method to predict B-cell linear antigenic epitopes. Methods Mol Biol (Clifton NJ). (2020) 2131:299–307. doi: 10.1007/978-1-0716-0389-5_17, PMID: 32162263

[41] Heidarinia H Tajbakhsh E Rostamian M Momtaz H. Two peptides derivate from acinetobacter baumannii outer membrane protein K as vaccine candidates: A comprehensive in silico study. BMC Res Notes. (2023) 16:128. doi: 10.1186/s13104-023-06409-9, PMID: 37391796 PMC10314613

[42] Vita R Blazeska N Marrama D Duesing S Bennett J Greenbaum J. The immune epitope database (Iedb): 2024 update. Nucleic Acids Res. (2025) 53:D436–d43. doi: 10.1093/nar/gkae1092, PMID: 39558162 PMC11701597

[43] Li Y He J Bao XJ Qiu QC Yuan XN Xu C. A study on allele frequencies and mismatching proportion of hla-a, B, cw, drb1 and dqb1 on high-resolution donor-recipient typing in chinese han population. Zhonghua yi xue yi Chuan xue za zhi = Zhonghua yixue yichuanxue zazhi = Chin J Med Genet. (2011) 28:92–8. doi: 10.3760/cma.j.issn.1003-9406.2011.01.021, PMID: 21287519

[44] Beskow AH Josefsson AM Gyllensten UB. Hla class ii alleles associated with infection by hpv16 in cervical cancer in situ. Int J Cancer. (2001) 93:817–22. doi: 10.1002/ijc.1412, PMID: 11519043

[45] Ghaderi M Nikitina L Peacock CS Hjelmström P Hallmans G Wiklund F. Tumor necrosis factor a-11 and dr15-dq6 (B*0602) haplotype increase the risk for cervical intraepithelial neoplasia in human papillomavirus 16 seropositive women in northern Sweden. Cancer epidemiology Biomarkers prevention: Publ Am Assoc Cancer Research cosponsored by Am Soc Prev Oncol. (2000) 9:1067–70., PMID: 11045789

[46] Butsashvili M Kajaia M Kochlamazashvili M Zarandia M Gagua T Meskhishvili D. Genotypic distribution of hpv among women of reproductive age in Georgia. Georgian Med News. (2016) 258):40–3., PMID: 27770526

[47] Ropón-Palacios G Chenet-Zuta ME Otazu K Olivos-Ramirez GE Camps I. Novel multi-epitope protein containing conserved epitopes from different leishmania species as potential vaccine candidate: integrated immunoinformatics and molecular dynamics approach. Comput Biol Chem. (2019) 83:107157. doi: 10.1016/j.compbiolchem.2019.107157, PMID: 31751887

[48] van den Hende M Redeker A Kwappenberg KM Franken KL Drijfhout JW Oostendorp J. Evaluation of immunological cross-reactivity between clade A9 high-risk human papillomavirus types on the basis of E6-specific cd4+ Memory T cell responses. J Infect Dis. (2010) 202:1200–11. doi: 10.1086/656367, PMID: 20822453

[49] Condie D. Roitt's Essential Immunology – 10th Edition [Book Review]. The Australian Journal of Medical Science. (2003) 24:212.

[50] Madeleine MM Johnson LG Smith AG Hansen JA Nisperos BB Li S. Comprehensive analysis of hla-a, hla-B, hla-C, hla-drb1, and hla-dqb1 loci and squamous cell cervical cancer risk. Cancer Res. (2008) 68:3532–9. doi: 10.1158/0008-5472.Can-07-6471, PMID: 18451182 PMC2662593

[51] Barlow DJ Edwards MS Thornton JM. Continuous and discontinuous protein antigenic determinants. Nature. (1986) 322:747–8. doi: 10.1038/322747a0, PMID: 2427953

[52] Chenzhang Y Wen Q Ding X Cao M Chen Z Mu X. Identification of the impact on T- and B- cell epitopes of human papillomavirus type-16 E6 and E7 variant in southwest China. Immunol Lett. (2017) 181:26–30. doi: 10.1016/j.imlet.2016.09.013, PMID: 27693214

[53] Sela-Culang I Ofran Y Peters B. Antibody specific epitope prediction-emergence of a new paradigm. Curr Opin Virol. (2015) 11:98–102. doi: 10.1016/j.coviro.2015.03.012, PMID: 25837466 PMC4456244

[54] Larsen JE Lund O Nielsen M. Improved method for predicting linear B-cell epitopes. Immunome Res. (2006) 2:2. doi: 10.1186/1745-7580-2-2, PMID: 16635264 PMC1479323

[55] Yao B Zhang L Liang S Zhang C. Svmtrip: A method to predict antigenic epitopes using support vector machine to integrate tri-peptide similarity and propensity. PloS One. (2012) 7:e45152. doi: 10.1371/journal.pone.0045152, PMID: 22984622 PMC3440317

[56] Rammensee H Bachmann J Emmerich NP Bachor OA Stevanović S. Syfpeithi: database for mhc ligands and peptide motifs. Immunogenetics. (1999) 50:213–9. doi: 10.1007/s002510050595, PMID: 10602881

[57] He J Li Q Ma S Li T Chen Y Liu Y. The polymorphism analysis and epitope predicted of alphapapillomavirus 9 E6 in sichuan, China. Virol J. (2022) 19:14. doi: 10.1186/s12985-021-01728-4, PMID: 35057815 PMC8772103

[58] Mobini Kesheh M Shavandi S Azami J Esghaei M Keyvani H. Genetic diversity and bioinformatic analysis in the L1 gene of hpv genotypes 31, 33, and 58 circulating in women with normal cervical cytology. Infect Agents Cancer. (2023) 18:19. doi: 10.1186/s13027-023-00499-7, PMID: 36959610 PMC10037780

