# Supplementary material for: Integrating bioinformatics to explore HPV-31 and HPV-52 E6/E7 proteins: from structural analysis to antigenic epitope prediction
Source: Front Immunol. 2025 Jul 25;16:1561572. doi: 10.3389/fimmu.2025.1561572 (PMC12333388; doi:10.3389/fimmu.2025.1561572)
Supplement: Supplementary Table 1 — Prediction results of ABCpred B cell epitopes of HPV-31 E6 protein. [file Table1.docx]

Supplementary Material

**Supplementary Tables**

**Table 1.** Prediction results of ABCpred B cell epitopes of HPV-31 E6 protein.

| **Rank** | **Sequence** | **Start position** | **Score** |
| --- | --- | --- | --- |
| 1 | DLTIVYRDDTPYGVCT | 49 | 0.91 |
| 2 | FHNIGGRWTGRCIVCW | 125 | 0.89 |
| 3 | YGVCTKCLRFYSKVSE | 60 | 0.89 |
| 3 | GRCIVCWRRPRTETQV | 134 | 0.89 |
| 4 | YGTTLEKLTNKGICDL | 84 | 0.85 |
| 5 | KVSEFRWYRYSVYGTT | 72 | 0.83 |
| 6 | DLLIRCITCQRPLCPE | 98 | 0.80 |
| 6 | ALEIPYDELRLNCVYC | 18 | 0.80 |

**Table 2.** Prediction of ABCpred B cell epitopes of HPV-31 E7 protein.

| **Rank** | **Sequence** | **Start position** | **Score** |
| --- | --- | --- | --- |
| 1 | EQLPDSSDEEDVIDSP | 26 | 0.92 |
| 2 | EEDVIDSPAGQAKPDT | 34 | 0.85 |
| 3 | RGETPTLQDYVLDLQP | 2 | 0.82 |
| 4 | AGQAKPDTSNYNIVTF | 42 | 0.80 |
| 5 | DLQPEATDLYCYEQLP | 14 | 0.79 |

**Table 3.** Prediction results of ABCpred B cell epitopes of HPV-52 E6 protein.

| **Rank** | **Sequence** | **Start position** | **Score** |
| --- | --- | --- | --- |
| 1 | MGRWTGRCSECWRPRP | 129 | 0.91 |
| 1 | TPLCPEEKERHVNANK | 108 | 0.91 |
| 2 | EERVRKPLSEITIRCI | 89 | 0.90 |
| 2 | YGVCIMCLRFLSKISE | 60 | 0.90 |
| 3 | YSLYGKTLEERVRKPL | 81 | 0.84 |
| 4 | DLRIVYRDNNPYGVCI | 49 | 0.83 |
| 5 | CEVLEESVHEIRLQCV | 16 | 0.79 |
| 5 | ERHVNANKRFHNIMGR | 116 | 0.79 |
| 6 | KKELQRREVYKFLFTD | 34 | 0.74 |

**Table 4.** Prediction of ABCpred B cell epitopes of HPV-52 E7 protein.

| **Rank** | **Sequence** | **Start position** | **Score** |
| --- | --- | --- | --- |
| 1 | HCYEQLGDSSDEEDTD | 23 | 0.90 |
| 2 | IVTYCHSCDSTLRLCI | 56 | 0.86 |
| 3 | ATSNYYIVTYCHSCDS | 50 | 0.85 |
| 3 | DTDGVDRPDGQAEQAT | 36 | 0.85 |
| 4 | GDSSDEEDTDGVDRPD | 29 | 0.78 |
| 5 | KATIKDYILDLQPETT | 5 | 0.75 |

**Table 5.** Prediction results of the Bepipred 1.0 Server B cell epitopes of HPV-31 E6 protein.

| **Rank** | **Sequence** | **Score** |
| --- | --- | --- |
| 1 | PRTETQV(143-149) | 1.032 |
| 2 | KNPAERPRK(3-11) | 0.9473 |
| 3 | RDDTPYG(55-61) | 0.8044 |
| 4 | LCPEEKQRHL(110-119) | 0.7805 |

**Table 6.** Prediction of the Bepipred 1.0 Server B cell epitopes of HPV-31 E7 protein.

| **Rank** | **Sequence** | **Score** |
| --- | --- | --- |
| 1 | QLPDSSDEEDVID  SPAGQAKPDTSNY(27-52) | 1.157 |
| 2 | MRGETPTL(1-8) | 1.086 |
| 3 | QPEAT(16-20) | 0.5584 |

**Table 7.** Prediction of the Bepipred 1.0 Server B cell epitopes of HPV-52 E6 protein.

| **Rank** | **Sequence** | **Score** |
| --- | --- | --- |
| 1 | MFEDPATRPR(1-10) | 1.033 |
| 2 | LCPEEKERHVN(110-120) | 0.8765 |
| 4 | CWRPRPVTQV(139-148) | 0.6947 |

**Table 8.** Prediction of the Bepipred 1.0 Server B cell epitopes of HPV-52 E7 protein.

| **Rank** | **Sequence** | **Score** |
| --- | --- | --- |
| 1 | LGDSSDEEDTDGVDRP  DGQAEQATS(28-52) | 1.776 |
| 2 | MRGDKAT(1-7) | 0.741 |
| 4 | QPETTD(16-21) | 0.621 |

**Table 9.** Prediction results of BCPreds B cell epitopes for HPV-31 E6 protein.

| **Rank** | **Sequence** | **Score** |
| --- | --- | --- |
| 112 | PEEKQRHLDKKKRFHNIGGR | 0.892 |
| 48 | TDLTIVYRDDTPYGVCTKCL | 0.804 |

**Table 10.** Prediction of BCPreds B cell epitopes for HPV-31 E7 protein.

| **Rank** | **Sequence** | **Score** |
| --- | --- | --- |
| 29 | PDSSDEEDVIDSPAGQAKPD | 0.999 |
| 79 | LQELLMGSFGIVCPNCSTRL | 0.938 |
| 4 | ETPTLQDYVLDLQPEATDLY | 0.787 |

**Table 11.** Prediction of BCPreds B cell epitopes for HPV-52 E6 protein.

| **Rank** | **Sequence** | **Score** |
| --- | --- | --- |
| 104 | IICQTPLCPEEKERHVNANK | 0.982 |
| 4 | DPATRPRTLHELCEVLEESV | 0.971 |
| 48 | TDLRIVYRDNNPYGVCIMCL | 0.932 |
| 129 | MGRWTGRCSECWRPRPVTQV | 0.915 |
| 25 | EIRLQCVQCKKELQRREVYK | 0.864 |

**Table 12.** Prediction of BCPreds B cell epitopes for HPV-52 E7 protein.

| **Rank** | **Sequence** | **Score** |
| --- | --- | --- |
| 29 | GDSSDEEDTDGVDRPDGQAE | 0.998 |
| 68 | RLCIHSTATDLRTLQQMLLG | 0.942 |
| 8 | IKDYILDLQPETTDLHCYEQ | 0.869 |

**Table 13.** Prediction of SVMTRIP B cell epitopes of HPV-31 E6 protein.

| **Rank** | **Sequence** | **Score** |
| --- | --- | --- |
| 30-49 | CVYCKGQLTETEVLDFAFTD | 1.000 |
| 97-116 | CDLLIRCITCQRPLCPEEKQ | 0.438 |
| 123-142 | KRFHNIGGRWTGRCIVCWRR | 0.341 |

**Table 14.** Prediction of SVMTRIP B cell epitopes of HPV-31 E7 protein.

| **Rank** | **Sequence** | **Score** |
| --- | --- | --- |
| 8-27 | LQDYVLDLQPEATDLYCYEQ | 1.000 |
| 58-77 | CCQCESTLRLCVQSTQVDIR | 0.684 |

**Table 15.** Prediction of SVMTRIP B cell epitopes of HPV-52 E6 protein.

| **Rank** | **Sequence** | **Score** |
| --- | --- | --- |
| 100-119 | TIRCIICQTPLCPEEKERHV | 1.000 |
| 122-141 | NKRFHNIMGRWTGRCSECWR | 0.601 |
| 74-93 | SEYRHYQYSLYGKTLEERVR | 0.587 |

**Table 16.** Prediction of SVMTRIP B cell epitopes of HPV-52 E7 protein.

| **Rank** | **Sequence** | **Score** |
| --- | --- | --- |
| 11-30 | YILDLQPETTDLHCYEQLGD | 1.000 |
| 66-85 | TLRLCIHSTATDLRTLQQML | 0.509 |

**Table 17.** CD4+T cell epitope prediction of HPV-31 E6 protein with HLA-DRB1*1501 as allele parameter.

|  | **SYFPEITHI** | | | **IEDB** | | |
| --- | --- | --- | --- | --- | --- | --- |
|  | **Position** | **Sequence** | **Score** | **Position** | **Sequence** | **Score** |
| HLA-DRBI 1501 | 64 | TKCLRFYSKVSEFRW | 26 | 64 | TKCLRFYSKVSEFRW | 3.10 |
|  | 39 | ETEVLDFAFTDLTIV | 24 | 63 | CTKCLRFYSKVSEFR | 3.40 |
|  | 101 | IRCITCQRPLCPEEK | 24 | 62 | VCTKCLRFYSKVSEF | 5.70 |
|  | 119 | LDKKKRFHNIGGRWT | 24 | 65 | KCLRFYSKVSEFRWY | 8.90 |
|  | 134 | GRCIVCWRRPRTETQ | 24 | 66 | CLRFYSKVSEFRWYR | 9.40 |

**Table 18.** Prediction of CD4+T cell epitopes of HPV-31 E7 protein using HLA-DRB1*1501 as allele parameter.

|  | **SYFPEITHI** | | | **IEDB** | | |
| --- | --- | --- | --- | --- | --- | --- |
|  | **Position** | **Sequence** | **Score** | **Position** | **Sequence** | **Score** |
| HLA-DRBI 1501 | 81 | ELLMGSFGIVCPNCS | 30 | 76 | IRILQELLMGSFGIV | 7.10 |
|  | 19 | ATDLYCYEQLPDSSD | 26 | 75 | DIRILQELLMGSFGI | 7.50 |
|  | 5 | TPTLQDYVLDLQPEA | 24 | 73 | QVDIRILQELLMGSF | 8.00 |
|  | 80 | QELLMGSFGIVCPNC | 24 | 70 | QSTQVDIRILQELLM | 8.50 |
|  | 51 | NYNIVTFCCQCESTL | 20 | 72 | TQVDIRILQELLMGS | 8.60 |

**Table 19.** Prediction of CD4+T cell epitopes of HPV-52 E6 protein with HLA-DRB1*1501 as allele parameter.

|  | **SYFPEITHI** | | | **IEDB** | | |
| --- | --- | --- | --- | --- | --- | --- |
|  | **Position** | **Sequence** | **Score** | **Position** | **Sequence** | **Score** |
| HLA-DRBI 1501 | 126 | HNIMGRWTGRCSECW | 28 | 64 | IMCLRFLSKISEYRH | 3.70 |
|  | 39 | RREVYKFLFTDLRIV | 24 | 63 | CIMCLRFLSKISEYR | 7.20 |
|  | 101 | IRCIICQTPLCPEEK | 24 | 65 | MCLRFLSKISEYRHY | 8.00 |
|  | 119 | VNANKRFHNIMGRWT | 24 | 71 | SKISEYRHYQYSLYG | 9.00 |
|  | 28 | LQCVQCKKELQRREV | 20 | 73 | ISEYRHYQYSLYGKT | 9.40 |

**Table 20.** Prediction of CD4+T cell epitopes of HPV-52 E7 protein with HLA-DRB1*1501 as allele parameter.

|  | **SYFPEITHI** | | | **IEDB** | | |
| --- | --- | --- | --- | --- | --- | --- |
|  | **Position** | **Sequence** | **Score** | **Position** | **Sequence** | **Score** |
| HLA-DRBI 1501 | 19 | TTDLHCYEQLGDSSD | 26 | 75 | ATDLRTLQQMLLGTL | 13.00 |
|  | 5 | KATIKDYILDLQPET | 24 | 76 | TDLRTLQQMLLGTLQ | 14.00 |
|  | 53 | NYYIVTYCHSCDSTL | 20 | 72 | HSTATDLRTLQQMLL | 17.00 |
|  | 75 | ATDLRTLQQMLLGTL | 20 | 73 | STATDLRTLQQMLLG | 18.00 |
|  | 82 | QQMLLGTLQVVCPGC | 20 | 74 | TATDLRTLQQMLLGT | 18.00 |

**Table 21.** Prediction of CE8+T cell epitopes of HPV-31 E6 oncogene using alleles HLA-A*1101 and HLA-A*0201 as parameters.

| **IEDB** | **Position** | **Sequence** | **Percentile rank** |
| --- | --- | --- | --- |
| HLA-A*1101 | 82-90 | SVYGTTLEK | 0.01 |
|  | 86-94 | TTLEKLTNK | 0.01 |
|  | 80-90 | RYSVYGTTLEK | 0.04 |
|  | 72-80 | KVSEFRWYR | 0.08 |
|  | 85-94 | GTTLEKLTNK | 0.09 |
| HLA-A*0201 | 11-19 | KLHELSSAL | 0.06 |
|  | 52-62 | IVYRDDTPYGV | 0.51 |
|  | 11-21 | KLHELSSALEI | 0.82 |
|  | 45-53 | FAFTDLTIV | 0.93 |
|  | 42-50 | VLDFAFTDL | 0.97 |

**Table 22.** Prediction of CE8+T cell epitopes of HPV-31 E7 oncogene using alleles HLA-A*1101 and HLA-A*0201 as parameters.

| **IEDB** | **Position** | **Sequence** | **Percentile rank** |
| --- | --- | --- | --- |
| HLA-A*1101 | 37-46 | VIDSPAGQAK | 0.33 |
|  | 89-97 | IVCPNCSTR | 1.9 |
|  | 36-46 | DVIDSPAGQAK | 2.0 |
|  | 44-52 | QAKPDTSNY | 3.1 |
|  | 85-97 | GSFGIVCPNCSTR | 3.4 |
| HLA-A*0201 | 7-15 | TLQDYVLDL | 0.09 |
|  | 11-19 | YVLDLQPEA | 0.15 |
|  | 66-74 | RLCVQSTQV | 0.95 |
|  | 82-90 | LLMGSFGIV | 1.1 |
|  | 11-22 | YVLDLQPEATDL | 1.5 |

**Table 23.** Prediction of CE8+T cell epitopes of HPV-52 E6 oncogene using alleles HLA-A*1101 and HLA-A*0201 as parameters.

| **IEDB** | **Position** | **Sequence** | **Percentile rank** |
| --- | --- | --- | --- |
| HLA-A*1101 | 86-94 | KTLEERVRK | 0.01 |
|  | 85-94 | GKTLEERVRK | 0.19 |
|  | 47-55 | FTDLRIVYR | 0.5 |
|  | 82-91 | SLYGKTLEER | 0.8 |
|  | 82-94 | SLYGKTLEERVRK | 0.88 |
| HLA-A*0201 | 18-26 | VLEESVHEI | 0.03 |
|  | 45-53 | FLFTDLRIV | 0.06 |
|  | 82-92 | SLYGKTLEERV | 0.12 |
|  | 11-19 | TLHELCEVL | 0.43 |
|  | 18-28 | VLEESVHEIRL | 0.48 |

**Table 24.** Prediction of CE8+T cell epitopes of HPV-52 E7 oncogene using alleles HLA-A*1101 and HLA-A*0201 as parameters.

| **IEDB** | **Position** | **Sequence** | **Percentile rank** |
| --- | --- | --- | --- |
| HLA-A*1101 | 51-59 | TSNYYIVTY | 0.74 |
|  | 50-59 | ATSNYYIVTY | 0.83 |
|  | 90-98 | MLLGTLQV | 2.5 |
|  | 87-98 | GTLQVVCPGCAR | 2.6 |
|  | 75-83 | ATDLRTLQQ | 2.8 |
| HLA-A*0201 | 84-92 | MLLGTLQVV | 0.08 |
|  | 83-91 | QMLLGTLQV | 0.17 |
|  | 11-19 | YILDLQPET | 0.5 |
|  | 84-91 | MLLGTLQV | 0.92 |
|  | 83-92 | MLLGTLQV | 0.99 |

**Table 25.** Prediction of HPV-31 E6 antigen dominant epitopes in T cells and B cells.

| **Episode** | **Methods** | **Location** | **Sequence** |
| --- | --- | --- | --- |
| B-cell epitope | ABCpred | 55-61 | RDDTPYG |
|  | BepiPred, | 112-116 | PEEKQ |
|  | BCPREDS | 125-131 | FHNIGGR |
|  | SVMTriP |  |  |
| T-cell epitope | SYFPEITHI | 45-53 | FAFTDLTIV |
|  | IEDB | 72-80 | KVSEFRWYR |

**Table 26.** Prediction of HPV-31 E7 antigen dominant epitopes in T cells and B cells.

| **Episode** | **Methods** | **Location** | **Sequence** |
| --- | --- | --- | --- |
| B-cell epitope | ABCpred | 8-17 | QDYYLDLQP |
|  | BepiPred, | 16-20 | QPEAT |
|  | BCPREDS | 29-41 | PDSSDEEDVIDEP |
|  | SVMTriP | 42-48 | AGQAKPDT |
| T-cell epitope | SYFPEITHI | 7-15 | TLQDYVLDL |
|  | IEDB | 82-90 | LLMGSFGIV |
|  |  | 11-19 | YVLDLQPEA |

**Table 27.** Prediction of HPV-52 E6 antigen dominant epitopes in T cells and B cells.

| **Episode** | **Methods** | **Location** | **Sequence** |
| --- | --- | --- | --- |
| B-cell epitope | ABCpred | 110-119 | LCPEEKERHV |
|  | BepiPred, | 129-141 | MGRWTGRCSECWR |
|  | BCPREDS |  |  |
|  | SVMTriP |  |  |
| T-cell epitope | SYFPEITHI | 45-53 | FLFTDLRIV |
|  | IEDB | 82-87 | SLYGKT |

**Table 28.** Prediction of HPV-52 E7 antigen dominant epitopes in T cells and B cells.

| **Episode** | **Methods** | **Location** | **Sequence** |
| --- | --- | --- | --- |
| B-cell epitope | ABCpred | 11-19 | YILDLQPET |
|  | BepiPred, | 23-27 | HCYEQ |
|  | BCPREDS | 29-38 | GDSSDEEDTD |
|  | SVMTriP | 36-48 | DTDGVDRPDGQAE |
| T-cell epitope | SYFPEITHI | 84-90 | MLLGTLQ |
|  | IEDB | 11-19 | YILDLQPET |
|  |  | 53-59 | NYYIVTY |
